# A Method for Broadband Polyimide Permittivity Measurement of Silicon Interposer Applied for High Speed Digital Microsystem

**DOI:** 10.3390/mi13071138

**Published:** 2022-07-18

**Authors:** Zhuoyue Zheng, Yongkun Wang, Lei Han, Daowei Wu, Defeng Mo, Wenchao Tian

**Affiliations:** 1 Key Laboratory of Electronic Equipment Structure Design, Xidian Univercity, Xi’an 710071, China; zhuoyuezheng@stu.xidian.edu.cn (Z.Z.); leihan1005@163.com (L.H.); wctian@xidian.edu.cn (W.T.); 2 Xi’an Institute of Microelectronic Technology, Xi’an 710065, China; wudaowei1220@163.com; 3 Key Laboratory of Infrared Imaging Materials and Devices, Chinese Academy of Sciences, Shanghai 200000, China; dfmo@mail.sitp.ac.cn

**Keywords:** micro-system, interposer, polyimide, dielectric properties, microstrip line, iterative inversion method

## Abstract

High-speed digital microsystems has emerged as one of the most important solutions for improving system performance, bandwidth, and power consumption. Based on mature micro-system processing technology, a material extraction approach for silicon interposer applied for high-speed digital microsystems was presented in order to obtain frequency-dependent precise material parameters. By combining microwave theory and mathematical model of iterative algorithm, the dielectric constant (Dk) and the dissipation factor (Df) of polyimide dielectric layer is acquired, which minimizes testing costs and streamlines testing process. The method is based on two-port transmission/reflection measurements. Vector Network Analyzer (VNA) is used to extract the scattering parameters with an extraction range of 1 MHz to 10 GHz. The algorithm is programmed using MATLAB. The observed Dk values at 2 GHz, 6 GHz, 8 GHz, and 10 GHz are, respectively, 3.22, 3.04, 2.96, 3.03, and 2.91, while the corresponding Df values are 0.021, 0.025, 0.026, 0.026, and 0.024. Finally, the complex permittivity derived is simulated and analyzed using Ansys HFSS. The results verify the validity of the theoretical method and proves that the values of the complex permittivity obtained by the method in this paper are reliable.

## 1. Introduction

With the constant promotion of new infrastructures such as 5G and artificial intelligence, it is difficult to meet future development expectations just by enhancing system functionality and performance through process dimension reduction, single-chip area increase, and so on [1,2]. Microsystem technology, as an advanced integration technology that can overcome the limits of Moore’s Law, has emerged as one of the most important solutions for improving system performance, bandwidth, and power consumption [3].

As micro-systems develop more integrated, redistribute layer (RDL), a core Interposer technology, enables connectivity between integrated circuits. Although RDL is a mature process, the actual electrical characteristics of the interconnect may deviate from their expected behavior when the device operates at a higher frequency band, such as the current common Double Data Rate Synchronous Dynamic Random Access Memory (DDR SRAM) chipset system’s maximum operating frequency of gigahertz, resulting in reflection, loss, distortion, and other signal quality issues [4,5].

According to transmission line theory, the dielectric constant of the substrate material has a significant impact on signal integrity. As a result, understanding the precise dielectric constant and the trend of its frequency change is critical for the design, modeling, and studies. Although the manufacturer may provide raw material parameters, it is gradually unable to meet the needs of impedance control precision and signal propagation characteristics researchdue to limited frequency, inherent precision error in machining, and changes in storage and use environment.

A wide variety of dielectric materials are studied to meet requirements for desired electrical performance of digital microsystem [6]. Among them, polyimide is the most widely applied. Polyimide (PI) shows excellent mechanical and thermal properties, and high adhesion strength [7]. Therefore, it is worthwhile to investigate how to extract the Complex permittivity of PI based on the microsystem production process. The extraction of dielectric properties has been studied by a number of research works, and mostly used in the field of PCB substrate materials. The methods applied to interposer technology on micro-system are rarely reported. Measurement methods can be loosely classified into two categories: resonant and transmission/reflection [8]. The split-post dielectric resonator (SPDR) technique [9,10,11,12] is widely used for low-loss materials, but its test frequency is limited by the resonant frequency, and the material under test must be in the shape of a plane substrate with specific dimensions and thickness, whereas transmission/reflection technique measurement system is simple and straightforward to use, and is particularly well suited to measurements over a large frequency range [13,14]. The precision is slightly lower than the resonance approach with low loss materials [15].

Some authors in the literautre proposed unique design for the transmission line, which enhances precision of measurement. Narayanan [16] proposed a method consists of the measurement of the two-port scattering parameters of a microstrip line with an obstacle on three equidistant positions over this line. The method can minimize the effect of connector non-reproducibility and impedance mismatch problems. However, it is sensitive to obstacle positioning errors and is not recommended for use below 4 GHz. Krzysztof Szostak [17] used a single microstrip line with an adjustable width and proposed a new variant of the self-calibration method. The method was tested for low-loss materials in the frequency range of 1–8 GHz. The estimated error is under ±5% with reference to the SPDR method.

Without modifying the transmission line’s structure, some other approaches for extracting the dielectric constant have been offered. Huber [18] utilized various transmission line structures including microstrip and co-planar waveguide ground (CPWG). A proper empirical formula was provided, and considered several effects (surface waves, frequency dependent conductance, inductance, and resistance) for obtaining the attenuation constant of the line and the dielectric constant (Dk)of the substrate.The method’s validity is demonstrated by measurements of two types of RF-substrate materials up to 110 GHz. However, it is difficult to apply a large amount of theoretical computation to real engineering output. Based on Huber’s theoretical study, Huang et al. [19] proposed an method can also extract material parameters at 1 GHz to 110 GHz frequencies by using probe head to connect to frequency extenders. Comparisons are drawn between results acquired by strip line and microstrip line. However, the extraction precision is not high, especially the Dk value extracted by microstrip line, and only the change trend with frequency is given without quantitative analysis.

On the basis of the aforementioned research, this paper proposed a method for broadband polyimide permittivity measurement of silicon interposer applied for high-speed digital microsystems. This approach combines the search algorithm with the iterative inversion method to quickly extract the frequency variation trend of the material parameters while inheriting the benefits of the two-line method. It is based on the mature production process of wafer-level packaging and does not require any special fixtures. In comparison to the previous extraction method, it can provide a more budget—friendly design, reduce testing expenses, simplify the testing process, and be adjusted for speed and precision according to engineering requirements.

The algorithm requires scattering parameters and the physical size of the microstrip line as inputs. By combining microwave theory and transmission line theory, the Dk and Df of polyimide are extracted from 0.01 to 10 GHz. Scattering parameters is extracted by Vector Network Analyzer (VNA), and MATLAB is used to programmatically implement iterative inversion process. Proposed mothod is supported by the results of simulations and tests. The calibration error, connector repeatability, and impedance matching are the key drawbacks of the transmission reflection method. The short-open-load-thru (SOLT) approach is used to calibrate the probe-to-microstrip line transition. In addition, the transmission result is de-embedded using the multi-line approach in this study to ensure the precision and repeatability.

## 2. Methodology

The mathematical relationship between the relative permittivity and equivalent relative permittivity of a material is established using transmission line theory and microwave theory. The impact of dispersion cannot be ignored because devices in High-speed digital microsystems may frequently operate at gigahertz. The dispersive behavior of surface modes for microstrip line and equivalent line width was compensated using closed-form expressions.

VNA was used to extract the scattering parameters of the transmission line, equivalent relative permittivity of the material under the actual test structure can be derived [20]. Comparing the theoretical and practical equivalent permittivity. By modifying the parameters (Dk) in the model, the two values are gradually approximated by iteration until the acceptable error is reached (under which the two values are considered equal). Finally, output all Dk that meet the error requirements.

The method of modifying parameters is to first determine the range of possible distribution of Dk. An algorithm can be used to quickly obtain the Dk parameter range of the material to be tested at a small cost. According to the experimental data and results, the value of Dk will fluctuate with the change of frequency and range of Dk is continuous and narrow. An algorithm that can lock down the possible distribution of Dk value is constructed based on the characteristics of the fluctuation of Dk with frequency. The followings are the specific operation steps.

Step One: Set a wide range ([n,m]) according to the material category, for reference, the semiconductor material extracted in the article is Polyimide, set the Dk range from 1 to 10.

Step Two: Create a [10×10×2] 3-dimensional matrix, the first layer (i, j, 1) is used to store the 100 values in order, the second layer (i, j, 2) is used to store the result of the decision.

**Figure 1:**
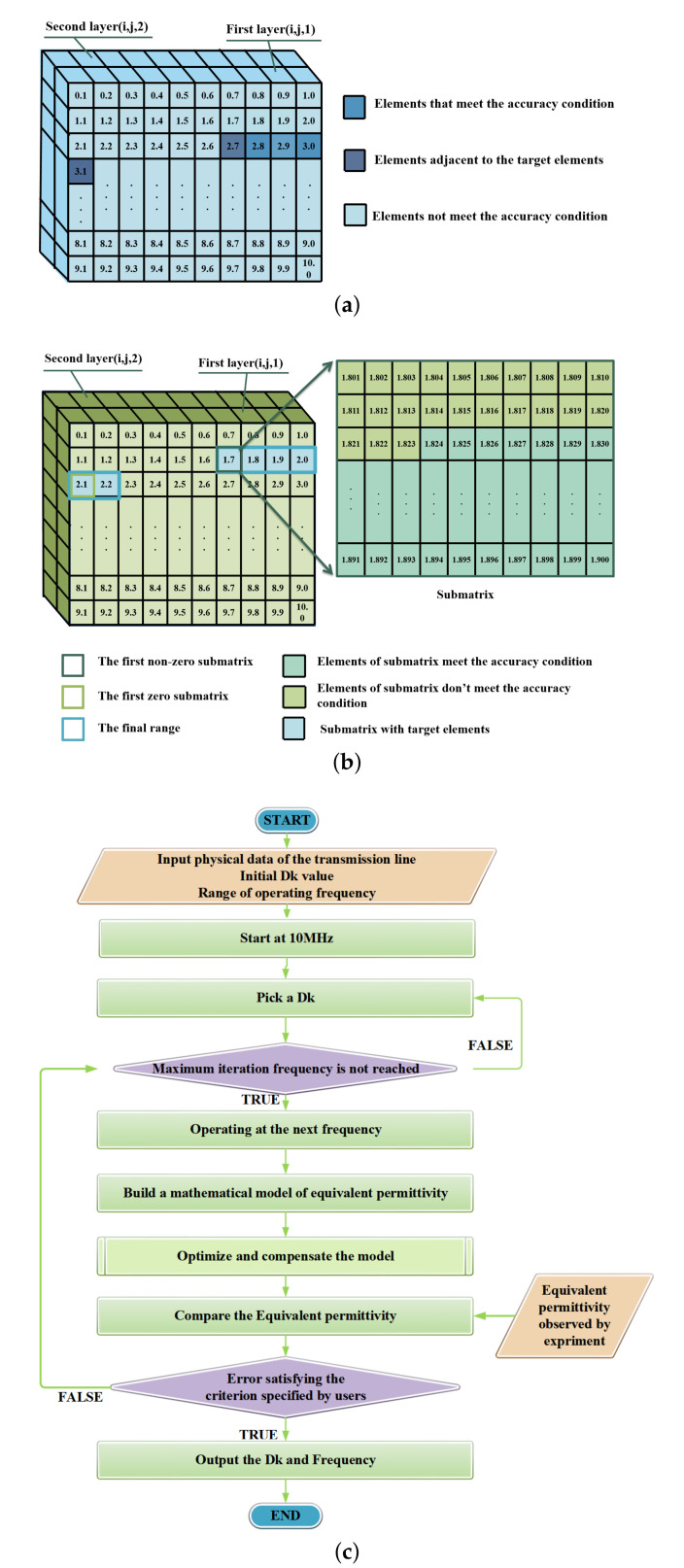
Schematic of retrieval algorithm. (**a**) First coarse-grained retrieval matrix. (**b**) Diagram of secondary retrieval. (**c**) Decision process.

Step Three: At each frequency in the interest frequency band, use the matrix element in (i, j, 1) as Dk to calculate theoretical equivalent permittivity, comparing the theoretical and practical equivalent permittivity, if the error is acceptable, mark the value (i, j, 2) of the element is as 1; if not, mark as 0; The decision process is shown in Figure 1c.

Step Four: After traversing, the value of an element in the (i, j, 2) layer should be filled with 0 or 1. If the value of an element in the (i, j, 2) layer is 1, then the corresponding element in the (i, j, 1) layer and the adjacent elements of that (i, j, 1) layer element are taken as the final range, as shown in Figure 1a.

Step Five: If the (i, j, 2) is a zero matrix, take the difference between elements as the initial range [n,m],then repeat step two to acquire the sub-matrices.

Step Six: Iterate the first layer elements of the sub-matrices, until the first element in sub-matrix is found that meets the permissible error, regard (1, 1, j) of this sub-matrix as the floor range of Dk. Continue to traverse until the first (i, j, 2) is found to be zero, regard the last element (100, 100, 1) of that matrix as the roof, as shown in Figure 1b.

Step Seven: If all second layer (i, j, 2) of the sub-matrices are zero matrices after traversal, repeat steps five to seven until the range boundaries are searched.

In order to avoid omitting the real Dk value when using this method, the decision accuracy should be set to a reasonable value. The real Dk range is regarded as a subset of the algorithm’s output range.

To verify the reliability of the range-retrieval algorithm, some of the most important factors that influence Dk value are investigate. Different dopants and preparation procedures will result in different Dk values for Polyimide. As listed in Table 1, the Dk value of Polyimide is generally distributed within 2.2 to 3.2 [21,22,23,24,25]. In the experiment of this paper, when the accuracy is set to 0.1, the Dk value retrieved by the algorithm is distributed within the range of 2.680 to 4.500, which proves the reliability of the range retrieval algorithm and the initial range suitable for iterative inversion is determined.

After obtaining the range of possible Dk distributions, a refined scope search within the range is performed using narrowed search step and permissible error, i.e., when the theoretical value of the equivalent dielectric constant calculated by Dk is close enough to the measured value, the Dk value is considered to be the dielectric constant of the material at that frequency. In this paper, the Dk is extracted with a search step of 0.001 and a verdict error of 0.01.

## 3. Theory and Calculation

### 3.1. The Equivalent Permittivity Obtained by Microstrip Line

#### 3.1.1. Fabrication

The procedure for determining the material’s dielectric constant requires obtaining the scattering characteristics of the microstrip line and using the observed data to calculate the equivalent permittivity.Instead of using a coaxial wire to link the two ports of the VNA, a probing technique is employed to obtain more trustworthy measurement data.

**Figure 2:**
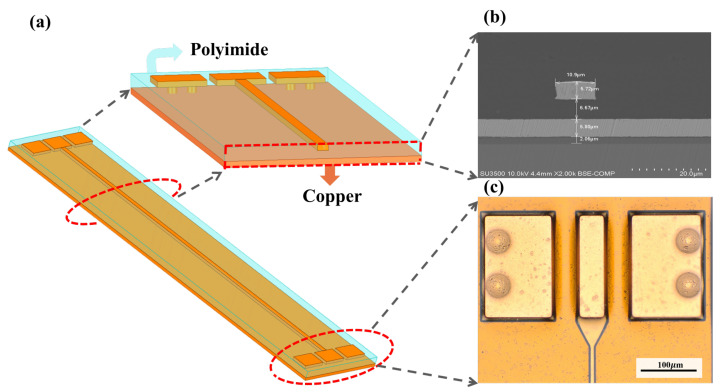
(**a**) 3D schematic diagram of the microstrip line. (**b**) Scanning electron microscope (SEM) photos of the test structure’s cross section. (**c**) Probe pad structure under 20× microscope.

The measurement system includes a vector network (Keysight N5224B) and a probe table (Cascade PM300). Based on the micro-system process, the microstrip line used to extract parameters is designed.The test structure consists of two parts: probe pad and microstrip line. To match G-S-G probe head, probe pads are fabricated with the width of 60 μm, pitch of 10 μm, and the length of 200 μm. Two vias are placed under each probe pad and connected to the ground layer. The scattering parameters were measured using VNA with a 10 MHz to 10 GHz bandwidth and 1000 points. The microstrip line is constructed with a substrate thickness of 8 μm, a metal thickness of 5 μm, and a signal strip width of 10 μm. Three different microstrip line with lengths of 12, 22, and 32 mm were developed, as shown in Figure 2. In the process of taping, X-ray Fluorescence Spectrometer is used to test the process parameters of the transmission line at 5 points,as shown in Table 2.

Five samples were taken from the top, middle, bottom, left and right regions of the wafer for measurement. The redundancy in test structures enables a variance analysis within the measured values and can verify the accuracy and repeatability of the extraction method.

#### 3.1.2. Calibration and Measurement

The effective dielectric constant can be deduced in the quasi-static analysis by:

(1) $$\varepsilon_{eff}\overset{\mathrm{def}}{=}\frac{c}{v_{p}}=\left[\mathrm{imag}\left(\gamma \right)c/\omega \right]$$

*C* is the speed of light in free space, and $v_{p}$ is the phase velocity of a signal transmission, γ is the propagation constant of a transmission line. In order to determine the effective dielectric constant of the material, we need to measure the scattering parameters of microstrip line scattering parameters, so as to obtain the accurate γ of the transmission line.

**Figure 3:**
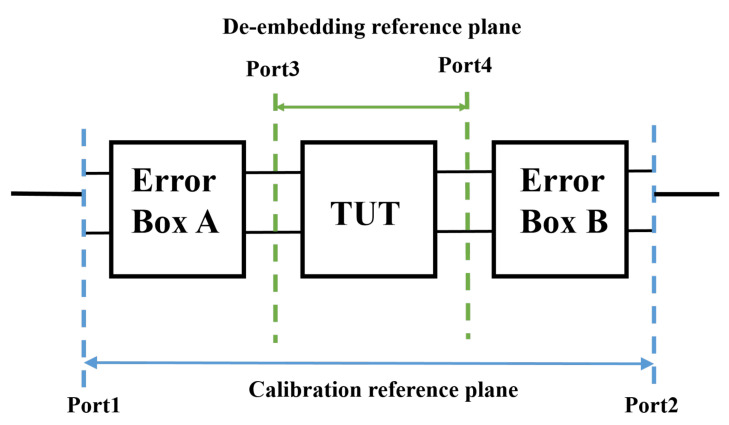
Schematic diagram of calibration and de-embedding.

When performing measurements, the influence of system error is not negligible. In addition, using the transmission and reflection method, inaccuracies and impedance mismatches generated by repetitive connector operation will have a significant impact on the results, so it is necessary to calibrate the measurement system in advance. In order to eliminate the internal error of vector network analyzer (including cable and probe), firstly, using the standard calibration parts (short, open, load and thru ) of vector network analyzer for calibration. SOLT calibration method can provide excellent accuracy and repeatability. After calibration, the reference planes are between the port of 1 and 2, as shown in Figure 3.

Given that the transmission line has probe pads and other features, in order to obtain the S parameters of the two-port network with uniform lines, it is necessary to conduct further de-embedding operation on the measurement results. Multi-line method is selected for de-embedding [26] and the accuracy of the de-embedding method is discussed in [27].

The two de-embedding reference planes are between the port of 3 and 4, as shown in Figure 3. Error boxes represent the systematic errors, and transmission line under test (TUT) are set at locations on the lines where only dominant traveling waves propagate. The line segment between the reference planes can thus be modeled as a matched line, irrespective of the actual value of the characteristic impedance [28]. The scattering parameter matrix $\left[S_{ij}\right]$ and the transmission matrix $\left[T_{ij}\right]$ have the following conversion relationship.

(2) $$\left[\begin{array}{cc} T_{11} & T_{12}\\ T_{21} & T_{22} \end{array}\right]=\frac{1}{S_{21}}\left[\begin{array}{cc} S_{21}S_{12}-S_{11}S_{22} & S_{11}\\ -S_{22} & 1 \end{array}\right]$$

Define $\left[T_{i}\right]$ as wave cascade matrices transformed from measured scattering parameter. [$L_{i}$] represents the TUT of different lengths,[$T_{L}]$ and [$T_{R}$] represents the error boxes. [$T_{1}$] and [$T_{2}$] can be expressed by

(3) $$\left[T_{1}\right]=\left[T_{L}\right]\left[L_{1}\right]\left[T_{R}\right]$$

(4) $$\left[T_{2}\right]=\left[T_{L}\right]\left[L_{2}\right]\left[T_{R}\right]$$

The systematic errors include the contact impedance, impedance mismatch and errors introduced by testable design (PAD). For all measurements, the error matrices are the same and can be eliminated during the calculation [16]. Equations (3) and (4) can be transformed to

(5) $$\left[T_{1}\right]{\left[T_{2}\right]}^{-1}=\left[T_{L}\right]\left[L_{1}\right]{\left[L_{2}\right]}^{-1}{\left[T_{L}\right]}^{-1}$$

Assumed that the reference planes to be merged at its middle plane. Subsequently, the following eigen equation can be formed by:

(6) $$\left[T_{1}\right]{\left[T_{2}\right]}^{-1}=\left[T_{L}\right]\left[\begin{array}{cc} e^{\gamma l_{i}} & 0\\ 0 & e^{-\gamma l_{i}} \end{array}\right]{\left[T_{L}\right]}^{-1}$$

$l_{i}$ represents the difference in length between lines. $l_{1}$ (10 mm) denotes the difference between a medium line (22 mm) and a shorter line (12 mm), whereas $l_{2}$ (20 mm) represents the difference between a longer line (32 mm) and a shorter line (12 mm). When the equality relation in (5) exists, according to the definition of matrix similarity, $\left[T_{1}\right]{\left[T_{2}\right]}^{-1}$, $\left[L_{1}\right]{\left[L_{2}\right]}^{-1}$ are same-order matrices, and [$T_{L}$] is invertible, the matrix $\left[T_{1}\right]{\left[T_{2}\right]}^{-1}$ is similar to $\left[L_{1}\right]{\left[L_{2}\right]}^{-1}$ and the eigenvalues of the two similar matrices are the same, so

(7) $$\mathrm{eig}\left(\left[T_{1}\right]{\left[T_{2}\right]}^{-1}\right)=\mathrm{eig}\left(\left[L_{1}\right]\left[L_{2}^{-1}\right]\right)=\lambda_{i},i=1,2;$$

For a similar matrix, the sum of the eigenvalues is equal to the sum of its diagonal elements, i.e.,

(8) $$\lambda_{1}+\lambda_{1}=tr\left(\left[T_{1}\right]{\left[T_{2}\right]}^{-1}\right)=tr\left(\left[T_{1}\right]{\left[T_{2}\right]}^{-1}\right)$$

$\left[L_{1}\right]{\left[L_{2}\right]}^{-1}$ can be expressed as:

(9) $$\left[L_{1}\right]{\left[L_{2}\right]}^{-1}=\left[\begin{array}{cc} e^{\gamma l_{i}} & 0\\ 0 & e^{-\gamma l_{i}} \end{array}\right]$$

(10) $$tr\left(\left[L_{1}\right]{\left[L_{2}\right]}^{-1}\right)=e^{\gamma l_{i}}+e^{-\gamma l_{i}}$$

Therefore, Equation (10) can be written as:

(11) $$\cosh\left(\gamma l\right)=\frac{\mathrm{tr}\left(\left[\left[T_{1}\right]*{\left[T_{2}\right]}^{-1}\right]\right)}{2}$$

(12) $$\gamma =\cosh^{-1}\left(\frac{\left|\left[T_{1}\right]*{\left[T_{2}\right]}^{-1}\right|}{2}\right)/l_{i}$$

where $tr\left(\left[T_{1}\right]{\left[T_{2}\right]}^{-1}\right)$ represent the trace and determinant of matrix and denote complex hyperbolic cosine functions. Through the Formulas (1)–(12), the equivalent permittivity of the unknown material can be obtained from the measured data. Then, readily remove the sign-assignment problem of square roots frequently occurring in complex propagation constant extractions.

### 3.2. Effective Permittivity Calculated by Theory

The dielectric property of the substrate is described by the permittivity as

(13) $$\varepsilon =\varepsilon \times \varepsilon_{0}=\left(\varepsilon^{\prime }-\varepsilon^{\prime \prime }j\right)\times \varepsilon_{0}$$

(14) $$\tan\delta =\frac{\varepsilon^{\prime }}{\varepsilon^{\prime \prime \prime }}$$

where $\varepsilon^{\prime }$ and tanδ are defined as the dielectric constant Dk and dissipation factor. The line width of the microstrip line is *w*, and the material thickness is *h*. When *w* is larger than *h*, the effective dielectric constant in the quasi-static analysis can be formulated by [29]:

(15) $$\varepsilon_{eff}=\frac{\varepsilon^{\prime }+1}{2}+\frac{\varepsilon^{\prime }-1}{2}{\left(1+12*\frac{h}{w}\right)}^{-1/2}$$

Considering the actual manufacturing size, the metal thickness and wire width are compensated based on microwave theory [29].

(16) $$\varepsilon_{eff_{\mathrm{thickmetal}}}=\varepsilon_{eff}-\frac{\left(\varepsilon^{\prime }-1\right)t}{4.6\sqrt{wh}}$$

(17) $$W_{eff}=w+\Delta$$

(18) $$\Delta =\left(1.25t\pi \right)\left[1+\ln\frac{2h}{t}\right]$$

There are various closed-form expressions available which predict the dispersive behavior of surface modes for microstrip line. Two contributions show high degrees of accuracy in [19,30], and for computational evaluation, both approaches depend only on normalized dimensions conductor width *w* and substrate height *h* of the manufactured dimensions. Numerical approaches and the simulation of these approaches were compared and little difference were found [18]. Formulas from [19] were chosen for the compensation.

### 3.3. Extraction of Df

The attenuation constant is the real part of the propagation constant. Without consider the effect of radiation loss, the attenuation constant can be approximated by conductor and dielectric losses. It is considered that there is no cross influence between each other in the case of small attenuation.

(19) $$\alpha \approx \alpha_{c}+\alpha_{d}$$

For a lossy microstrip line with w>h, the current on the metal conduction strip can be roughly assumed uniformly distributed along the width direction, hence the conductor loss can be approximately expressed as:

(20) $$\alpha_{c}\approx \frac{R_{S}}{W_{eff}*Z}$$

$R_{S}$ is the surface impedance of the metal guide strip of the microstrip line, is the equivalent width of the line, and *Z* is the characteristic impedance of the transmission line. The dielectric loss of microstrip lines can be described as a function of the Dk and the Df, so the Df can be deduced from known quantities [29].

(21) $$\alpha_{d}=\frac{2\pi f\varepsilon^{\prime }\left(\varepsilon_{eff}^{-1}\right)\tan\delta }{2c\sqrt{\varepsilon_{eff}}\left(\varepsilon^{\prime }-1\right)}$$

## 4. Results and Discussion

**Figure 4:**
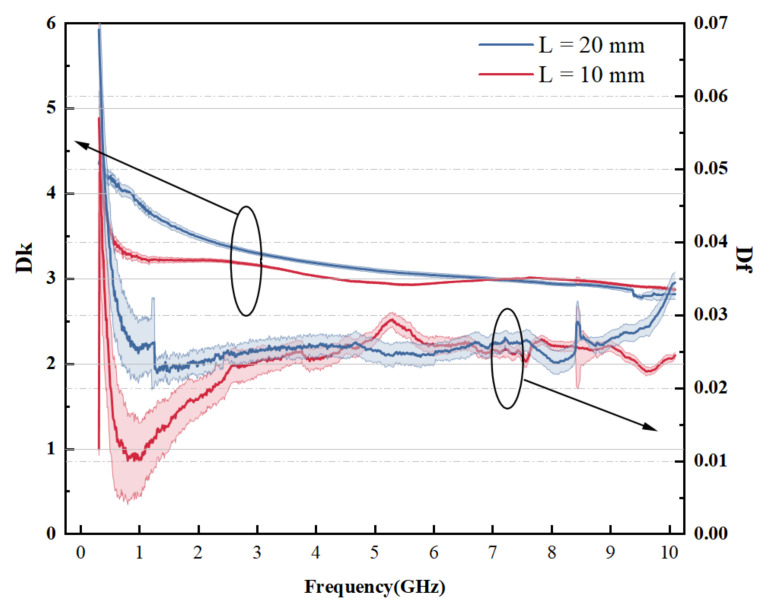
Measured Dk and Df of Polyimide for 10 mm and 20 mm Microstrip line (An ellipse and arrow are used to indicate the y-axis of the corresponding curve).

Figure 4 shows the derived Dk and Df value acquired and Table 3 shows the selected results of derived Dk and Df. Line length of 10 mm is shown in red, and 20 mm is shown in blue, and the error band is shown as a light-colored strip of the same color. Figure 4 shows that the Dk fluctuates with frequency, decreases slowly as the frequency increases. Except for the low frequency region, the Df shows no evident changing trend with increasing frequency (less than 1 GHz).

Both Dk and Df value have relatively large aberration under 1 GHz, and results produced by L=10 mm and L=20 mm also show little difference. A similar trend is observed for the measured extracted results in [2]. When using a differential phase length method [31], the size of the line affects the value of Dk and Df. This is due to the long wavelength of the low frequency band, One tenth wavelength may out long microstrip line within 1 GHz, resulting in inaccurate measurement results. Similarly, it is considered the difference in the relative length (10 mm and 20 mm) between the wavelength and microstrip line contributes to the difference. Wavelength decreases as the frequency increases. Line length has less of an impact on the outcomes, and the outcomes of the two lengths are typically comparable.

In addition, errors might be introduced in the measurement and manufacture. It may be another reason for the disparity between the Dk, Df values obtained for the two lengths. The error band in the figure depicts the standard deviation of data. Five sets of duplicate structures with three lengths of microstrip lines were fabricated, and several trials were carried out to ensure the repeatability of experiment. The standard deviation is for determining how much data values differ from the arithmetic mean [32]. It can be concluded from observation that the extracted value does not change much when the experiment is repeated. As a result, it is assumed that the experiment is repeatable.

**Figure 5:**
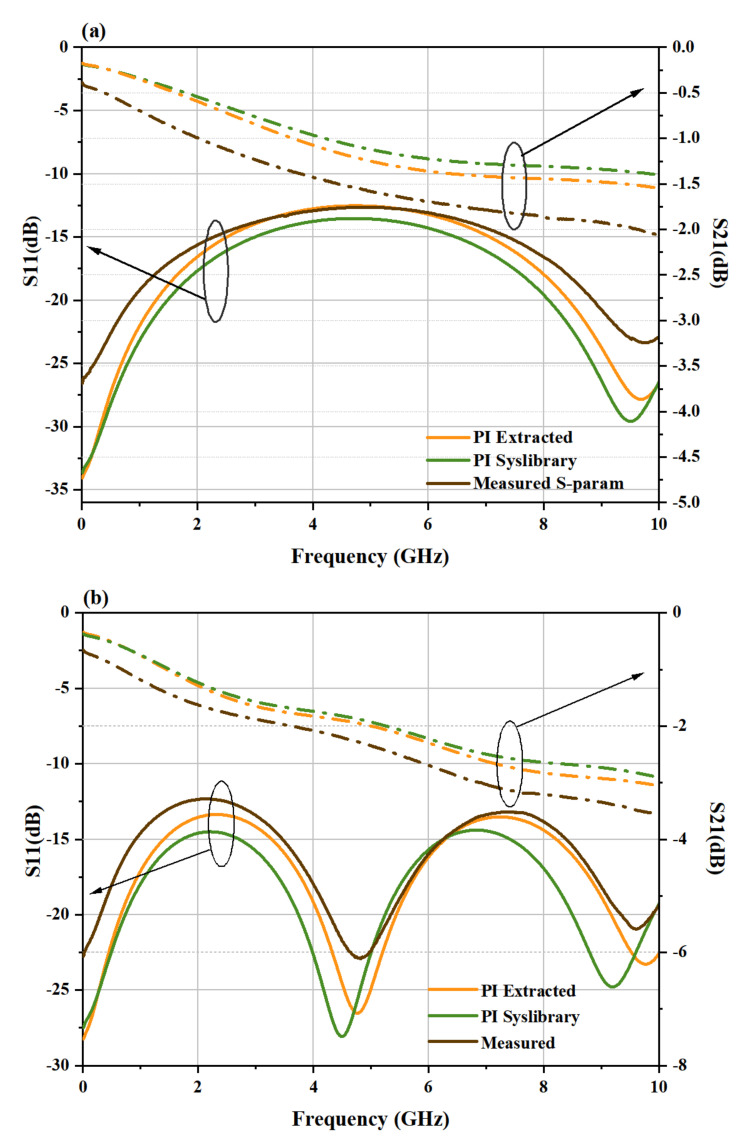
Schematic of retrieval algorithm. (An ellipse and arrow are used to indicate the y-axis of the corresponding curve). (**a**) Comparison between the test and simulation result of 10 mm. (**b**) Comparison between the test and simulation of 20 mm.

For proving that the Dk of the material extracted by this method is reliable, Ansys HFSS, a 3D electromagnetic simulation software, was used to simulate the microstrip line structure. In addition to adopting the same physical size as the test structure, derived dielectric constant and loss tangent obtained by microstrip lines of different lengths will also be separately used as the material parameters in HFSS modeling. Scattering parameter of test and simulation is compared. In the material library of HFSS, the Complex permittivity of the dielectric layer materials was 3.5, and the replacedDfDissipation factor was 0.008. Therefore, simulation was carried out using polyimide value in system library of HFSS (PIS) and Polyimide value extracted (PIE) above, respectively. In order to improve the simulation accuracy, broadband mode is used. The frequency is entered only when each frequency point within 0.01–10 GHz meets the convergence condition. Set the minimum convergence times to 5 to prevent false convergence. Finally, the simulation results in Figure 5 are obtained.

In general, compared to S-parameters derived with PIS, those obtained with PIE are more comparable and fit the measurement data better. The test results show that the transmission structure has good transmission characteristics, with return loss larger than −2.1 dB and insert loss less than −12.5 dB (10 mm). Figure 5a,b shows the return loss and insertion loss of microstrip lines of different lengths. It indicates that as the line length increases, the value of return loss increases while insert loss decreases, which implies that the signal has more reflection along the transmission line and for lossy conductors, line length is positively correlated with loss.

The difference in insertion loss between PIS and PIE is not particularly obvious, especially in the relatively low frequency region where the two curves almost overlap and deviate from the measured results.In fact, the loss characterized by the insertion loss includes a variety of losses such as dielectric loss, conductor loss, etc. Despite the fact that the parameter correction corrects the error caused by dielectric loss inaccuracy, the parameters of conductor surface roughness, metal conductivity, magnetic permeability, and process manufacturing may still be inaccurate. As a result, the overall change caused by the parameter correction is not dramatic. As the frequency increases, the rate of increase in dielectric loss is faster than that in attenuation caused by the wire, the proportion of dielectric loss in the total loss will increase, so the gap between the two parameters widens.

The PIS results differ more from the observed values, and the frequency interval between the curve’s peaks (or troughs) is smaller. This is owing to inaccuracy of Dk, which results in a lower impedance of the PIS-simulated microstrip line compared to the test structure. Because of the Dk value of PIS is larger, it cause the slower transmission rate and the transmission delay is greater on the same length than on the actual line, which leads to a larger phase difference at the same frequency. The phase difference affects the reflection coefficient, which leads to a smaller interval between the curve peaks. In contrast, the Dk value of the PIE is more accurate and its results closely match the measured curve. The average error of PIE is 5.53% while that of PIS is 13.70%. However manufacturing tolerances is still the main sources of uncertainty and may directly influence the S-parameters and so all subsequent computations. The measurement and simulation results shows the Complex permittivity obtained using the iterative inversion method are trustworthy. For the original data in the database of EDA simulation tools, the use of modified value can get more accurate simulation results.

## 5. Conclusions

This paper provides a method that enables the extraction of feature parameters applied to microsystem interposer materials and software implementation of the method. The Df and Dk of Polyimide at 0.01–10 GHz are extracted based on the microstrip structure. The scattering parameters of microstrip lines are measured by VNA. The short-open-load-thru approach is used to calibrate the probe-to-microstrip line transition. In addition, the transmission result is de-embedded using the multi-line approach. MATLAB is used to programmatically implement the iterative inversion process. Simulation and measurement are used to verify the validity of the retrieved parameters and methodologies. The method in the paper does not specify the material and can theoretically be applied to other semiconductor materials with Dk less than 20. In future works, the method described in this research can be used to derive Dk from additional materials. Although the line length is set as 10, 20 mm in order to obtain more accurate permittivity, valid parameters cannot be obtained in the low frequency range. The following issue aims to increase the low frequency band extraction accuracy. The characterisation of materials at high frequencies is becoming more important as 5G and high data-rate applications arise, and need to be investigated in order to retain accuracy while frequency band expanding.

## Figures and Tables

**Table 1:** Dielectric properties of polyimide.

Polyimide	Dk Values
ODA-PMDA	3.10 ± 0.006
Siloxane Polyimide	2.9
Rigid Polyimide	3.3
ODA-PMDA with different patterning method	3.0 (Wet Etch Laser)/2.7 (Laser) /2.7 (Photo-Litho. Laser)
ODA-PMDA with Different percentages of PM (polyimide microsphere)	2. 48 ± 0. 007 (10%)/2. 42 ± 0. 001 (20%) /2.30 ± 0.003 (40%)/2.26 ± 0.006 (50%)

**Table 2:** Process parameters of the transmission line.

Material	Design Value	Process Deviation
Copper	Thick = 5 μm	Within ±1.5 μm
	Width = 10 μm	Within ±0.5 μm
Polyimide	Thickness = 8 μm	Within ±3 μm

**Table 3:** Selected results of derived Dk and Df.

Frequency	2 GHz	4 GHz	6 GHz	8 GHz	4 GHz
Dk (L=10 mm)	3.22	3.04	2.96	3.03	2.91
Dk (L=20 mm)	3.49	3.15	3.04	2.94	2.82
Df (L=10 mm)	0.021	0.025	0.026	0.026	0.024
Df (L=20 mm)	0.023	0.026	0.025	0.024	0.033

## References

[1] Watanabe A.O., Ali M., Sayeed S., Rao R.T., Raj P.M. (2020). A Review of 5G Front-End Systems Package Integration. IEEE Trans. Compon. Packag. Manuf..

[2] Mahjabeen N., McIntyre H., Goel Y., Gowri S.P., Henderson R. (2019). Dielectric characterization of stacked packaging substrates using a coaxial transmission line technique. Proceedings of the 2019 IEEE Texas Symposium on Wireless and Microwave Circuits and Systems (WMCS).

[3] Wang Z. (2019). Microsystems using three-dimensional integration and TSV technologies: Fundamentals and applications. Microelectron. Eng..

[4] Lho D., Park H., Park S., Kim S., Kang H., Sim B., Kim S., Park J., Cho K., Song J. (2022). Channel Characteristic-Based Deep Neural Network Models for Accurate Eye Diagram Estimation in High Bandwidth Memory (HBM) Silicon Interposer. IEEE Trans. Electromagn. Compat..

[5] Bogatin E. (2010). Signal and Power Integrity—Simplified.

[6] Liu F., Zhang R., De Prospo B.H., Dwarakanath S., Nimbalkar P., Ravichandran S., Weyers D., Kathaperumal M., Tummala R.R., Swaminathan M. Advances in High Performance RDL Technologies for Enabling IO Density of 500 IOs/mm/layer and 8-*μ*m IO Pitch Using Low-k Dielectrics. Proceedings of the 2020 IEEE 70th Electronic Components and Technology Conference (ECTC).

[7] Dong X., Zheng M.S., Zha J.W. Low-*k* cross-linked polyimide for microelectronic packaging application. Proceedings of the 2021 International Conference on Electrical Materials and Power Equipment (ICEMPE).

[8] Hasar U.C., Westgate C.R. (2009). A Broadband and Stable Method for Unique Complex Permittivity Determination of Low-Loss Materials. IEEE Trans. Microw. Theory Tech..

[9] Heinola J.M., Tolsa K. (2006). Dielectric characterization of printed wiring board materials using ring resonator techniques: A comparison of calculation models. IEEE Trans. Dielectr. Electr. Insul..

[10] Rautio J.C., Rautio B.J. High accuracy broadband measurement of anisotropic dielectric constant using a shielded planar dual mode resonator. Proceedings of the Microwave Measurement Symposium.

[11] Krupka J., Gregory A.P., Rochard O.C., Clarke R.N., Riddle B., Baker-Jarvis J. (2001). Uncertainty of complex permittivity measurements by split-post dielectric resonator technique. J. Eur. Ceram. Soc..

[12] Kumar A., Sharma S., Singh G. (2007). Measurement of Dielectric Constant and Loss Factor of the Dielectric Material at Microwave Frequencies. Prog. Electromagn. Res..

[13] Coonrod J. (2011). Understanding the variables of dielectric constant for PCB materials used at microwave frequencies. Proceedings of the 2011 41st European Microwave Conference.

[14] Lee M.Q., Nam S. (1996). An accurate broadband measurement of substrate dielectric constant. IEEE Microw. Guid. Wave Lett..

[15] Słobodzian P., Barteczka B., Golonka L., Macioszczyk J. A comparison of two practical methods for measurement of the dielectric constant of LTCC substrates. Proceedings of the International Conference on Microwaves.

[16] Narayanan P.M. (2014). Microstrip Transmission Line Method for Broadband Permittivity Measurement of Dielectric Substrates. IEEE Trans. Microw. Theory Tech..

[17] Szostak K., Sobodzian P. (2018). Broadband Dielectric Measurement of PCB and Substrate Materials by Means of a Microstrip Line of Adjustable Width. IEEE Microw. Wirel. Compon. Lett..

[18] Huber O., Faseth T., Magerl G., Arthaber H. (2018). Dielectric Characterization of RF-Printed Circuit Board Materials by Microstrip Transmission Lines and Conductor-Backed Coplanar Waveguides Up to 110 GHz. IEEE Trans. Microw. Theory Tech..

[19] Huang C.C., Peng C.L., Lin P.Y., Yang B.H., Cheng K.C., Fu W.T. Dielectric Characterization of Printed Circuit Board by Microstrip Line Up to 110 GHz. Proceedings of the Electrical Design of Advanced Packaging and Systems Symposium.

[20] Kobayashi M. (1988). A Dispersion Formula Satisfying Recent Requirements in Microstrip CAD. IEEE Trans. Microw. Theory Tech..

[21] Tahar S., Benabed F., Boudra S., Seghiour A. Dielectric Proprieties and R elaxation Behavior of Polyimide Films ( PI). Proceedings of the Conference Internationale en Sciences et Technologies Electriques au Maghreb.

[22] Fujiwara T., Tatsuta Y., Matsumura K., Kanamori D., Tomikawa M. Development of Low Dielectric Loss Polyimides and Fabrication of Advanced Packagings for 5 g ApplicationS. Proceedings of the 2020 International Wafer Level Packaging Conference (IWLPC).

[23] Araki H., Yu S., Masuda Y., Hashimoto K., Tomikawa M. Fabrication of Redistribution Structure Using Highly Reliable Photosensitive Polyimide for Fan Out Panel Level Packages. Proceedings of the 2018 International Wafer Level Packaging Conference (IWLPC).

[24] Qiu G., Ma W., Wu L. (2020). Low dielectric constant polyimide mixtures fabricated by polyimide matrix and polyimide microsphere fillers. Polym. Int..

[25] Araki H., Kiuchi Y., Shimada A., Ogasawara H., Tomikawa M. (2020). Low Df Polyimide with Photosensitivity for High Frequency Applications. J. Photopolym. Sci. Technol..

[26] Marks R.B. (1991). A multiline method of network analyzer calibration. IEEE Trans. Microw. Theory Tech.

[27] Moon S.J., Ye X., Smith R. Comparison of TRL calibration vs. 2x thru de-embedding methods. Proceedings of the 2015 IEEE Symposium on Electromagnetic Compatibility and Signal Integrity.

[28] Fuh K.F. (2013). Broadband Continuous Extraction of Complex Propagation Constants in Methods Using Two-Line Measurements. IEEE Microw. Wirel. Compon. Lett..

[29] Clarricoats P. (2001). Foundations for Microwave Engineering.

[30] Kirschning M., Jansen R.H. (1982). Accurate model for effective dielectric constant of microstrip with validity up to millimetre-wave frequencies. Electr. Lett..

[31] Chen L.F., Ong C.K., Neo C.P., Varadan V.V., Varadan V.K. (2004). Microwave Electronics: Measurement and Materials Characterization.

[32] Bure V., Parilina E. (2013). Probability Theory and Mathematical Statistics.

